# Spatiotemporal characteristics of environmental microbial communities and resistomes in intensive care units of a tertiary hospital in northwest China

**DOI:** 10.3389/fmicb.2026.1909107

**Published:** 2026-09-09

**Authors:** Jiexi Yan, Qingmiao Ren, Xiaomin He, Haiyun Du, Lijuan He, Zhaoyu Zheng, Yaya Qi, Xiaohong Xian

**Affiliations:** 1 The Precision Medicine Laboratory, The First Hospital of Lanzhou University, Lanzhou, Gansu, China; 2 The Clinical High-Throughput Sequencing Technology Center of Gansu Province, Lanzhou, Gansu, China; 3 BGI Genomics, Shenzhen, China

**Keywords:** healthcare-acquired infection, intensive care unit, microbial and ARG profiles, mNGS, spatiotemporal distribution

## Abstract

**Background:**

Intensive care units (ICUs) are high-risk settings for healthcare-acquired infections (HAIs). It is essential to characterize the microorganisms and antimicrobial resistance genes (ARGs) in ICU environments for prevention and control of HAIs.

**Methods:**

Totally, 206 environmental and patient samples were collected from general ICU (GICU), emergency ICU (EICU), pediatric ICU (PICU), and neonatal ICU (NICU). Longitudinal sampling was conducted in PICU at four time points from May 2024 to March 2025. All samples were subjected to metagenomic sequencing. The differences of microbial and ARG profiles were further analyzed across different ICU types as well as sampling areas and times.

**Results:**

Alpha diversity did not differ among ICU types. In contrast, microbial community composition varied across ICUs (PERMANOVA, *p* = 0.001). GICU and EICU samples clustered together, enriched with *Acinetobacter baumannii* and *Klebsiella pneumoniae*, whereas NICU and PICU samples showed greater similarity, with enrichment of *Streptococcus pneumoniae* and *Burkholderia cepacia*. Sampling areas also exhibited differences in both alpha diversity (*p* < 0.001) and beta diversity (*p* = 0.001). Similar spatial patterns were observed for ARGs. Although ARG alpha diversity remained relatively stable, beta diversity differed significantly among ICU types and sampling areas (*p* = 0.001). Temporal variation was also evident in the PICU, where both microbial and ARG profiles changed over time (*p* = 0.001), and clinically relevant pathogens reached their highest abundance in November.

**Conclusions:**

These findings demonstrate obvious spatial and temporal variability in microbial and ARG profiles across ICU environments, supporting the development of targeted infection control strategies.

## Introduction

Healthcare-acquired infections (HAIs) remain one of the public health challenges worldwide. Recent systematic reviews and meta-analyses have reported that in developing countries, the overall prevalence of HAI is approximately 22% (Odoom et al., 2025), while it reaches 34% among critically ill patients (Assis et al., 2022). By comparison, the overall prevalence of HAIs in China's general hospitals is estimated at 2.5%, which is significantly lower than the average level in developing countries (Liu et al., 2023). HAIs increases the risk of death for hospitalized patients, leading to heavy economic and psychological burden on both patients and public health (Lemiech-Mirowska et al., 2021). Nevertheless, primary healthcare facilities, resource-limited hospitals, and intensive care units (ICUs) remain priority areas for infection prevention and control in China.

The occurrence of HAIs is associated with various factors, including host immune status, pathogen pathogenicity, antimicrobial resistance, invasive surgeries, and the hospital environment (Lemiech-Mirowska et al., 2021; Kollef et al., 2021). Among these factors, the hospital environment has long been considered as an important reservoir and transmission medium for HAI-associated pathogens (Cruz-López et al., 2023; Monteiro et al., 2022). The environmental surfaces, medical equipments, airborne particles, and the healthcare workers May serve as critical vectors for transmitting pathogens (Chng et al., 2020). Existing evidences have demonstrated that pathogens colonization in the ICU environment is closely related to patient infections (Peters et al., 2022; Mazzeffi et al., 2021). Notably, several organisms can survive on environmental surfaces for a long time, which increasing the risk of cross-transmission (Peters et al., 2022; Mazzeffi et al., 2021). Classical microbiological investigations have identified key HAIs-related pathogens such as *Acinetobacter baumannii, Pseudomonas aeruginosa, Enterococcus faecalis, Klebsiella pneumoniae, Enterobacter species, and* methicillin-resistant *Staphylococcus aureus* (MRSA) as frequent contaminants in ICU environments (Chng et al., 2020; van der Schoor et al., 2023). These findings highlight the importance of microbial contamination in the environment for HAIs. However, the traditional pathogen detection methods, such as culture and serology, are unable to fully characterize the microbial community structure and the diversity of potential pathogens.

In recent years, high-throughput sequencing (HTS) technology has emerged as a powerful tool to comprehensively characterize microbial communities in the environments (Zhang et al., 2021; Cason et al., 2022). HTS allows researchers to detect unculturable or low-abundance microorganisms, and also explore complex interactions between pathogens and commensal species (Zhang et al., 2021; Cason et al., 2022). Several studies have investigated microbial compositions in hospital environments through HTS. Studies from Lax et al. (2017) and Brooks et al. (2017) have demonstrated microbial exchange among the hospital environments, patients, and healthcare workers using 16S rRNA sequencing. Two other studies focused on inanimate surfaces in adult ICU and neonatal ICU (NICU), and the results revealed unique microbial communities on surfaces closest to patients, with NICU exhibiting more extensive pathogen contamination than adult ICU (Ribeiro et al., 2019; Christoff et al., 2020). A recent investigation used 16S rRNA sequencing to dynamically monitor bacterial communities across two ICUs over 1-year period. Findings from this study revealed marked differences in composition and diversity between these two ICUs, indicating the importance of environmental microbial monitoring for HAI prevention (Li et al., 2022). Moreover, a 6-month longitudinal investigation conducted using metagenomic next-generation sequencing (mNGS) at a geriatric ICU revealed the highest microbial diversity and abundance in floors, as well as potential transfer of antimicrobial resistance genes (ARGs) among patients (Yang et al., 2023). However, current studies on the dynamics monitoring of hospital environmental microbiota in ICU remain limited in China.

Our study monitored microbial communities and ARGs profiles in multi-site environmental surfaces across the general ICU (GICU), emergency ICU (EICU), pediatric ICU (PICU), and NICU (neonatal ICU) over 1 year through mNGS. This study aimed to characterize the spatiotemporal differences of microbiota and ARGs in different ICU environments. Our study expected to provide scientific support for the formulation of targeted infection prevention measures and the optimization of environmental disinfection protocols.

## Material and methods

### Study design and sample collection

Samples were collected from the GICU, EICU, PICU, and NICU in May 2024. Longitudinal sampling was performed in the PICU at four time points spanning May 2024 to March 2025 in the First Hospital of Lanzhou University. All environmental samples were collected 3 h after routine morning ward disinfection. In each ICU, we randomly selected three wards and collected nine environmental samples, including door handle, water faucet, computer mouse/keyboard, floors, infusion pumps, monitors, medical carts, ventilator surfaces and air, from each ward. Samples from door handle, water faucet, mouse/keyboard, floor, and air were grouped as public-area samples. Those from medical cart, infusion pump, monitor, and ventilator were grouped as medical-device samples. Sampling was performed at four fixed time points (May 2024, August 2024, November 2024, and March 2025). During sampling, staff wore sterile gloves and wiped target surfaces for 2 min with sterile normal saline-pre-moistened swabs, and then rotated them to maximize contact area (at least 5 cm × 5 cm). Then, three swabs were collected from each site and pooled into a tube containing normal saline. Air samples were collected using disinfected air sampler for 20 min (Zhang et al., 2024). Patient sampling was performed in accordance with strict random sampling principles. During the study period, eligible hospitalized patients were randomly selected in the sampling ward. For each enrolled patient, paired nasal swabs and hand swabs were collected simultaneously by professional medical staff using standardized aseptic sampling procedures (World Health Organization, 2020). All ICU medical staff was unaware of the ongoing study during sampling and data collection. And a hospital-wide antibiotic stewardship program has been implemented at our institution, including routine antimicrobial use audits, participation of clinical pharmacists in ward rounds, and regular training for clinical staff to standardize rational antimicrobial use. All samples were immediately stored at −80 °C for mNGS. In addition, routine multidrug-resistant organism (MDRO) surveillance data were obtained from the hospital Infection Control Department for the corresponding study period. Surveillance indicators included methicillin-resistant *Staphylococcus aureus* (MRSA), carbapenem-resistant *Enterobacterales* (CRE), carbapenem-resistant *Acinetobacter baumannii* (CRAB), carbapenem-resistant *Pseudomonas aeruginosa* (CRPA), and vancomycin-resistant *Enterococcus* (VRE). The numbers of hospitalized patients and detected MDRO isolates were collected for the corresponding study period.

This study was approved by the Ethics Committee of the First Hospital of Lanzhou University (LDYYLL-2025-2047). All procedures followed the Declaration of Helsinki, and informed consent was obtained from the legal guardians of all participants.

### DNA extraction, library construction, and sequencing

DNA was extracted from 300 μL sample using nucleic acid extraction and purification kit (BGI Biotechnology, Wuhan, China), and then stored at −80 °C until use. A Qubit fluorometer (Thermo Fisher Scientific, Waltham, MA, USA) was used to quantify DNA concentration. Next, DNA was performed with end-repair, adapter ligation, and PCR amplification to construct library. The Agilent 2100 Bioanalyzer (Agilent Technologies, Santa Clara, California, USA) was used to assess the fragment size, and library concentration was measured using Qubit fluorometer (Thermo Fisher Scientific, USA). Subsequently, DNA nanoballs (DNBs) were prepared by rolling circle amplification (RCA) method, and loaded onto sequencing chips. The sequencing was conducted on the BGISEQ-200 platform for 100 bp paired-end.

### Bioinformatics analyses

Fastp (v0.20.1) was used to quality control for raw sequencing, including removing low-quality reads and adapter sequences. Clean reads were then mapped to the human reference genome (hg38) using HISAT2 (v2.2.1), and host-derived reads were removed. Next, potential ribosomal RNA (rRNA) sequences were filtered out using SortMeRNA (v4.3.6). Lastly, the high-quality reads were annotated using Kraken2 (v2.1.2) to profile the microbial community composition.

For the following downstream analysis, we exclude the samples with <10,000 reads and low-frequency species detected in <5% of all samples. We first rarefied all samples to a uniform sequencing depth to minimize bias. Alpha diversity was assessed using the Shannon index. Beta diversity was evaluated using principal coordinates analysis (PCoA) based on Bray–Curtis distance matrices. Subsequently, we selected top 20 and 30 most abundant genera, and then visualized by stacked bar plots and hierarchically clustered heatmaps, respectively. Furthermore, species correlation analysis was conducted based on Pearson correlation coefficients, and co-occurrence patterns were displayed using hierarchically clustered heatmaps. All analyses were performed in R (v4.2.3) utilizing the phyloseq, vegan, ggplot2, and pheatmap packages.

We also analyzed ARGs using the Resistance Gene Identifier (RGI, v6.0.3) and the Comprehensive Antibiotic Resistance Database (CARD, v4.0.0). Specifically, clean reads were aligned to the CARD database using the BWA algorithm, and then the alignments were parsed and filtered with the RGI bwt module to yield annotation results for ARGs. To obtain high-confidence ARG annotations, only ARG hits with an average percent coverage ≥80% and an average mapping quality (MAPQ) > 20 were retained for downstream analyses. ARG abundance was normalized as reads per kilobase per million mapped reads (RPKM). Alpha and beta diversity, as well as hierarchical clustering heatmap analyses of resistance gene profiles, were performed using the same methods as described for microbial community analysis.

### Statistical analysis

Statistical analyses were performed in R. Differences in alpha diversity among groups were assessed using the Kruskal–Wallis test, followed by Dunn's multiple-comparison test with Bonferroni correction. Adjusted *p* < 0.05 were considered statistically significant. Beta diversity significance based on Bray–Curtis distances was assessed using PERMANOVA, and multiple hypothesis testing was corrected by false discovery rate (FDR) adjustment, with significance defined as FDR-adjusted *p* < 0.05.

## Results

### Basic information of sample and sequencing data

**Figure 1:**
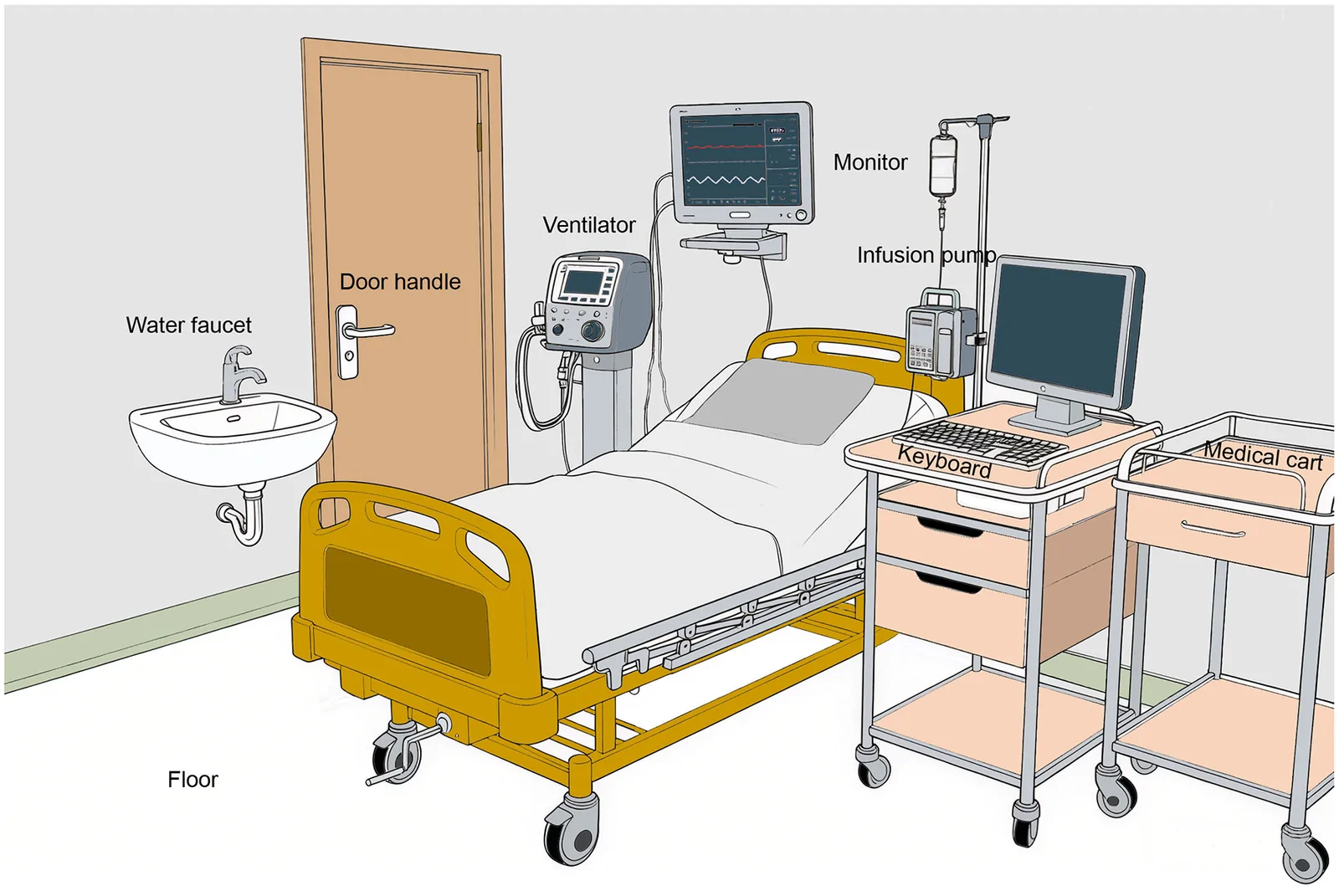
Overview of sampling sites.

We selected 4 ICUs and randomly chose 3 wards from each one. Then, we selected 9 sampling points in each ward (Figure 1). In total, 206 samples were collected from 4 different ICUs and subjected to mNGS. Specifically, 33 samples were from the GICU, 33 from the EICU, and 28 from the NICU, all collected in May 2024. The remaining 112 samples were collected from the PICU at the time points of May 2024, August 2024, November 2024, and March 2025. Totally, mNGS yielded 7,467,202,280 raw reads, with an average of 36,248,555 per sample.

### Microbial diversity and composition of four ICUs

**Figure 2:**
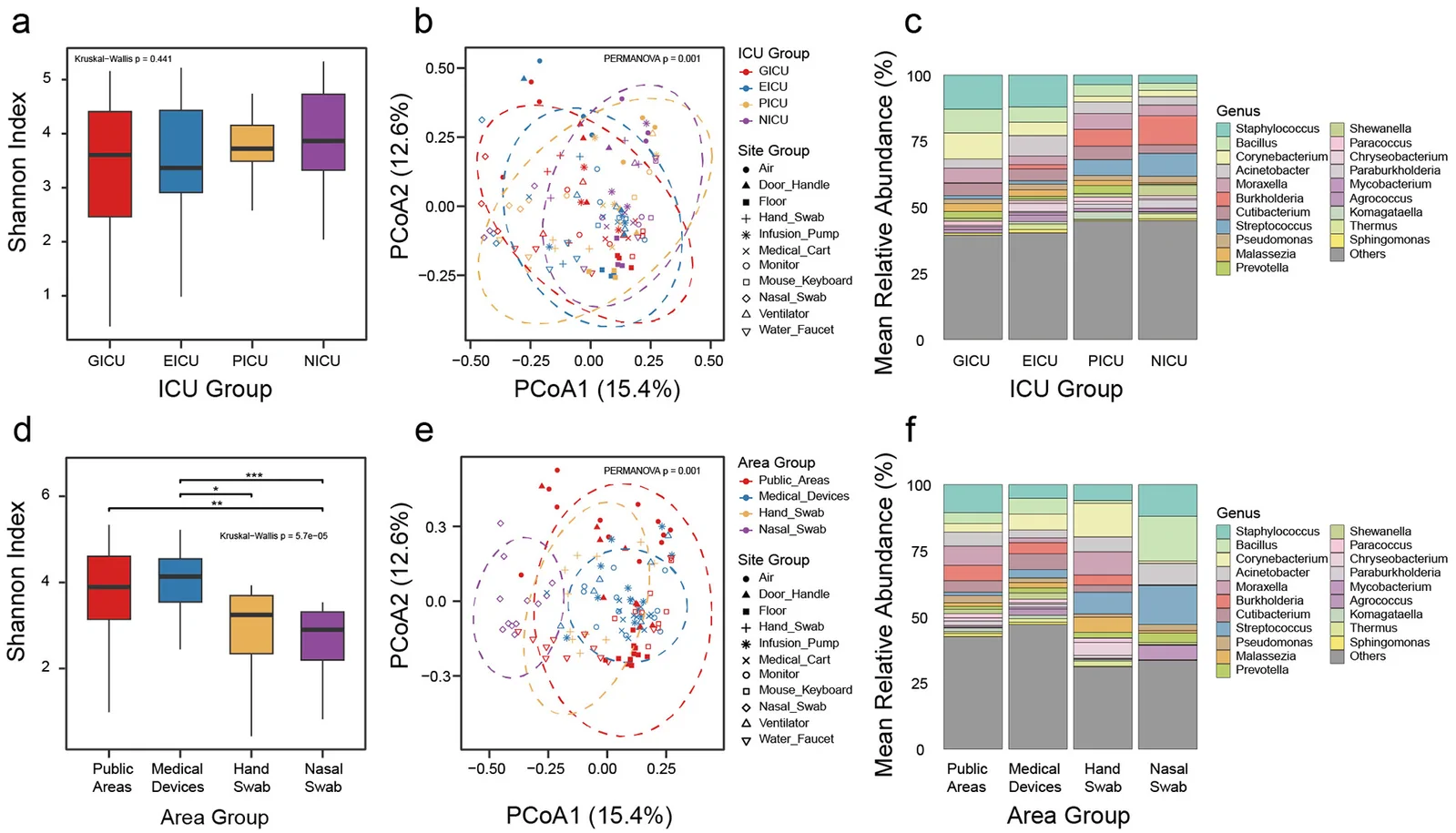
Microbial community characteristics at the genus level across different intensive care units (ICUs) type and sampling areas. **(a)** Alpha-diversity based on Shannon index in different ICUs, including general ICU (GICU), emergency ICU (EICU), pediatric ICU (PICU), and neonatal ICU (NICU). **(b)** Principal coordinates analysis (PCoA) of the beta diversity using Bray-Curtis distances in GICU, EICU, PICU, and NICU. **(c)** The mean relative abundances of top 20 genera in GICU, EICU, PICU, and NICU. **(d)** Alpha-diversity based on Shannon index in distinct ICU sampling areas (public areas, medical devices, nasal swabs, and hand swabs). **(e)** PCoA of the beta diversity using Bray-Curtis distances in distinct ICU sampling areas. **(f)** The mean relative abundances of top 20 genera in distinct ICU sampling areas. *p < 0.05, **p < 0.01, ***p < 0.001.

We first analyzed the overall microbiome profiles of different ICUs. Results from α diversity analysis showed that the Shannon indices of PICU and NICU samples were slightly elevated compared to GICU and EICU samples, while without statistically significant (Figure 2a). Our beta analysis revealed significant difference in microbial community across these ICUs based on PCoA with Bray-Curtis distances (*p* = 0.001, PERMANOVA, Figure 2b). Especially, a clear separation was observed between GICU/EICU and PICU/NICU (Figure 2b). The relative abundances of the top 20 genera further showed that *Staphylococcus, Corynebacterium*, and *Bacillus* exhibited high abundances in GICU and EICU, while PICU and NICU were dominated by *Burkholderia* and *Streptococcus* (Figure 2c).

We then analyzed the microbial characteristics across different sampling areas within each ICU. The results revealed significant difference in α diversity among public areas, medical devices, nasal swabs, and hand swabs (*p* < 0.001; Figure 2d). Among them, public areas and medical devices had similarly high α diversity, followed by hand swabs, and nasal swabs had the lowest diversity (Figure 2d). PCoA analysis also clearly revealed clustering of samples by sampling areas (*p* = 0.001, PERMANOVA), with nasal swabs showing distinct separation from the other three areas (Figure 2e). Taxonomic analysis of the top 20 genera (Figure 2f) further revealed that microbial composition patterns of environmental surfaces (public areas and medical devices) were highly similar, with both groups dominated by *Staphylococcus, Bacillus, Corynebacterium, Burkholderia*, and *Cutibacterium*. Hand swabs samples were characterized by a high abundance of skin-associated genera such as *Staphylococcus, Corynebacterium*, and *Streptococcu*s. Nasal swab samples were enriched with several opportunistic pathogens, including *Staphylococcus, Acinetobacter, Streptococcus*, and *Mycobacterium*. This finding clearly indicates region-specific microbial community structures.

Furthermore, we compared microbial community characteristics of the same sampling areas among different ICUs. The α diversity analysis revealed that in all ICUs, the Shannon indices of patient-derived samples (nasal and hand swabs) was lower than that of environmental surfaces (public areas and medical devices) (Supplementary Figures 1a–c). PCoA results (Supplementary Figures 1d–f) showed that for samples from public areas, GICU and EICU formed a tight cluster that was distinct from the PICU-NICU cluster. For medical equipment samples, the four ICUs exhibited a distinct overall separation, with a consistent subclustering pattern that grouped GICU and EICU together, as well as PICU and NICU into a separate cluster. Due to the limited sample size, no clear clustering pattern was observed for hand and nasal swab samples. Similarly, the relative abundances of the top 20 genera further confirmed the consistency in community composition between the GICU-EICU and PICU-NICU groups (Supplementary Figures 1g–i), which uncovered a two-group clustering patterns.

### Temporal dynamic patterns of microbial diversity and composition in the PICU over 1 year

**Figure 3:**
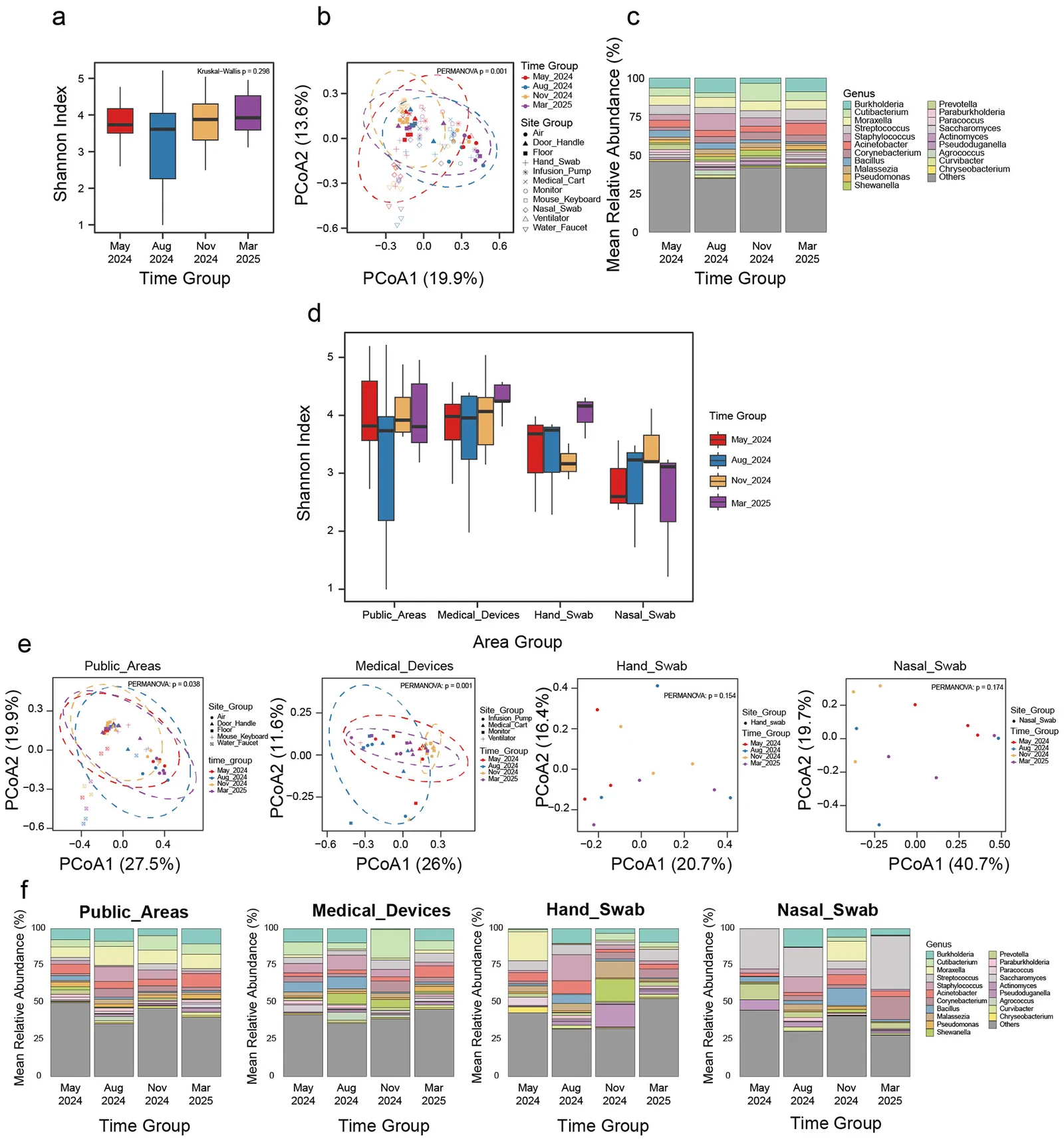
Microbial community characteristics of PICU at the genus level over a one-year period. **(a)** Alpha-diversity based on Shannon index of different sampling time-points in PICU. **(b)** PCoA of the beta diversity using Bray-Curtis distances of different sampling time-points in PICU. **(c)** The mean relative abundances of top 20 genera of different sampling time-points in PICU. **(d)** Alpha-diversity based on Shannon index at identical sampling areas across different sampling time-points within the PICU. **(e)** PCoA of the beta diversity using Bray-Curtis distances at identical sampling areas across different sampling time-points within the PICU. **(f)** The mean relative abundances of top 20 genera at identical sampling areas across different sampling time-points within the PICU.

Due to the PICU and NICU showing similar microbial compositions, we selected the PICU as a representative unit to investigate how its microbial community changed over the four sampling time points across the 1-year period (May 2024, August 2024, November 2024, and March 2025). The results found that there was no significant difference in α diversity among the four time points (Figure 3a). However, a distinct separation among different time groups was observed (PERMANOVA, *p* = 0.001, Figure 3b) using PCoA. The top 20 genera profiles also revealed that the relative abundance of several genera exhibited obvious changes across different sampling time points (Figure 3c). We found the highest relative abundance of *Burkholderia* in early autumn (August 2024) and the lowest in early winter (November 2024). The relative abundance of *Cutibacterium* was the lowest in early autumn and the highest in early winter. *Streptococcus* was more abundant during spring (March 2025) and summer (May 2024) but declined in autumn and winter. *Staphylococcus* reached its nadir in spring, progressively increased through summer, peaked in early autumn, and modestly declined in winter. *Acinetobacter* peaked sharply in spring while remaining relatively stable in other seasons. *Corynebacterium* displayed higher abundance in spring and winter but decreased during summer and autumn. Conversely, *Bacillus* was elevated during summer and autumn with progressive reduction through spring and winter. Subsequently, we further characterized temporal microbiome variations at identical sampling areas within the PICU. Specifically, alpha diversity analysis revealed no statistically significant differences in Shannon index across sampling timepoints, irrespective of sampling areas (Figure 3d). PCoA demonstrated statistically significant compositional segregation in both public areas (PERMANOVA, *p* = 0.038) and medical devices (PERMANOVA, *p* = 0.001) across different sampling timepoints, but not in patient-derived hand and nasal swabs (Figure 3e). Stacked bar plots of the top 20 genera demonstrated that for both environmental surfaces and patient-derived samples, some dominant opportunistic pathogenic microorganisms such as *Burkholderia, Streptococcus, Staphylococcus*, and *Acinetobacter* all exhibit temporal dynamic changes in abundance (Figure 3f).

### Spatiotemporal patterns of ICU microbial communities as revealed by hierarchical clustering and correlation networks

**Figure 4:**
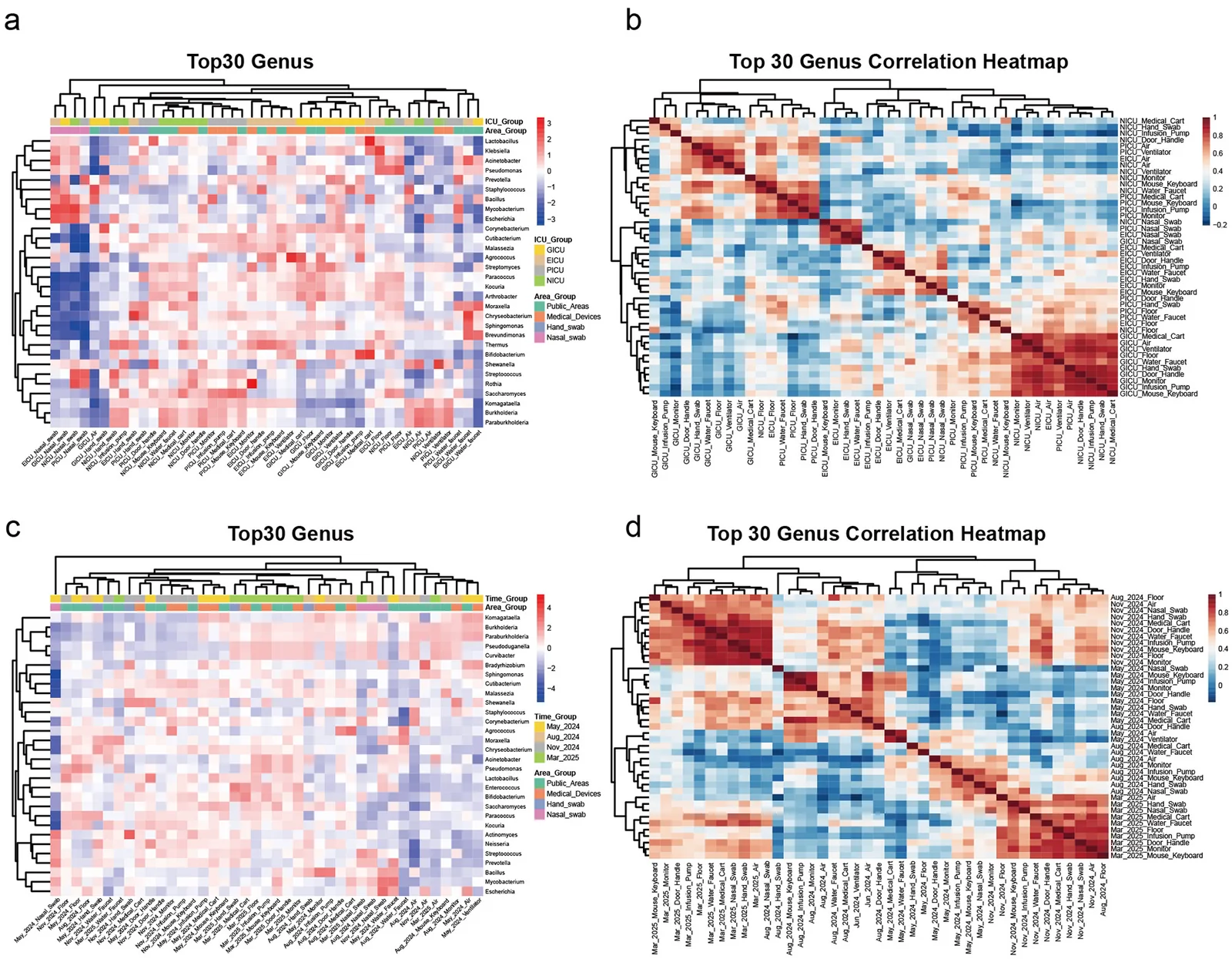
Spatiotemporal clustering and correlation patterns of ICU microbiota. **(a)** The hierarchical clustering heatmaps of top 30 genera across different ICUs and sampling areas. **(b)** The correlation heatmaps of top 30 genera across different ICUs. **(c)** The hierarchical clustering heatmaps of top 30 genera across different sampling time-points and sampling areas in PICU. **(d)** The correlation heatmaps of top 30 genera across different sampling time-points and sampling areas in PICU.

To further elucidate the spatiotemporal characteristics of microbial communities across different ICUs and at multiple time points within the PICU, we constructed hierarchical clustering heatmaps and correlation networks based on the top 30 genera. The hierarchical clustering heatmap (Figure 4a) revealed distinct microbial community structures across different ICUs and sampling sites. Consistent with previous findings, GICU and EICU samples clustered closely together, characterized by the enrichment of genera such as *Streptococcus, Saccharomyces, Burkholderia, Paraburkholderia, Rothia*, and *Komagataella*. NICU and PICU samples showed greater similarity, with enrichment of *Klebsiella, Pseudomonas, Corynebacterium*, and *Prevotella*. From the perspective of sampling areas, samples from the same area tended to cluster together. Samples from public areas were frequently clustered together with medical devices samples, while hand swabs and nasal swabs samples showed strong aggregation. Several common pathogenic genera, such as *Klebsiella, Acinetobacter, Staphylococcus, Bacillus, Mycobacterium*, and *Escherichia*, that highly enriched in nasal swabs were also found in various environmental samples. The correlation heatmap based on the top 30 genera (Figure 4b) revealed strong positive correlations among the samples from the same ICU, especially GICU, the relatively weak correlations were observed among different ICUs or sampling areas. Of note, floors and air samples displayed moderate correlations across different ICUs. Infusion pumps, door handles, and hand swabs also showed weak correlations within each ICU.

It was found that the microbial community structure of samples from the different time point exhibited temporal changes within the PICU by the hierarchical clustering heatmap (Figure 4c). We also observed increased relative abundance of several genera, such as *Komagataella, Burkholderia*, and *Paraburkholderia*, in May 2024 and August 2024. *Acinetobacter, Pseudomonas, Lactobacillus, Enterococcus, Bifidobacterium*, and *Saccharomyces* were obviously enriched in March 2025. The correlation heatmap results (Figure 4d) further found that samples collected at the same time point exhibited strong intra-group correlations, while correlations between samples from different time points were weak. Moreover, we found that floors and air maintained moderate correlations at different time points, suggesting that microbial communities exhibit a degree of temporal stability at these sampling areas.

### Spatiotemporal distribution of clinically relevant pathogens in the ICUs

**Figure 5:**
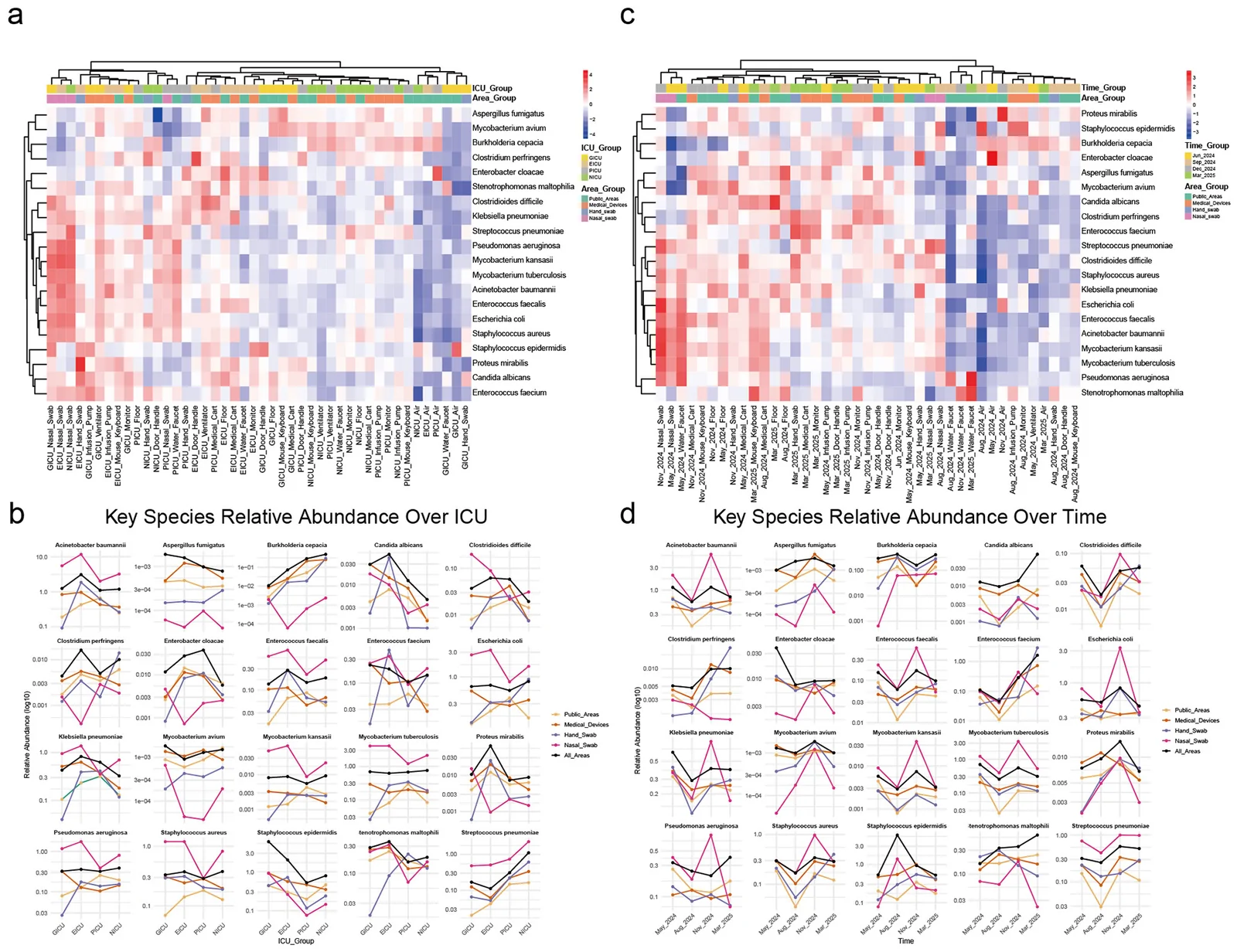
Distribution and abundance changes of healthcare-acquired infections (HAIs)-associated pathogens across ICUs. **(a)** The hierarchical clustering heatmaps of clinically relevant pathogens across different ICUs and sampling areas. **(b)** Relative abundance changes of clinically relevant pathogens across different ICUs and sampling areas. **(c)** The hierarchical clustering heatmaps of clinically relevant pathogens across different sampling time-points and sampling areas in PICU. **(d)** Relative abundance changes of clinically relevant pathogens across different sampling time-points and sampling areas in PICU.

Subsequently, we investigated the spatiotemporal distribution of 20 representative clinically relevant pathogens across different ICU types and multiple sampling areas. These pathogens were selected a priori based on published literature describing microorganisms commonly associated with HAIs, opportunistic infections, and severe respiratory diseases, including major nosocomial bacterial pathogens, opportunistic fungi, and clinically important mycobacterial species (Peters et al., 2022; Miller and Arias, 2024; Osman et al., 2024; Azim and Ahmed, 2024). Cluster heatmaps results (Figure 5a) showed that most key pathogens exhibited higher relative abundances in adult ICUs (GICU and EICU) compared to PICU /NICU. In addition, with respect to sampling areas, the relative abundance of key pathogens was relatively the highest in nasal swabs, while air samples were the lowest.

Further analysis demonstrated that clinically significant opportunistic pathogens—such as *Acinetobacter baumannii, Pseudomonas aeruginosa, Staphylococcus aureus, Staphylococcus epidermidis, Escherichia coli, Enterococcus faecalis, Enterococcus faecium, Clostridioides difficile, Klebsiella pneumoniae, Mycobacterium tuberculosis*, and *Streptococcus pneumoniae*—were consistently abundant across ICU samples, with similar levels in environmental and hand swabs, but peaking in nasal swabs (Figure 5b). Meanwhile, common fungal pathogens (*Candida albicans, Aspergillus fumigatus*) and environmental bacteria (*Burkholderia cepacia, Clostridium perfringens, Mycobacterium avium*) displayed lower abundances (Figure 5b).

With respect to ICU types, *Acinetobacter baumannii, Klebsiella pneumoniae, Enterococcus faecalis, Enterococcus faecium, Staphylococcus epidermidis*, and Proteus mirabilis were enriched in adult ICUs, while *Streptococcus pneumoniae* and *Burkholderia cepacia* were more abundant in pediatric ICUs (Figure 5b). However, several pathogens, such as *Pseudomonas aeruginosa, Staphylococcus aureus*, and *Escherichia coli*, exhibited similar distributions across ICU types (Figure 5b).

Cluster heatmaps results of clinically relevant pathogens in the PICU across four time points showed significant temporal dynamics in community composition (Figure 5c). Notably, pathogen abundances were higher in November 2024 and March 2025 compared with other periods. Line plots further demonstrated that that most key pathogens, such as *Acinetobacter baumannii, Clostridioides difficile, Enterococcus faecalis, Enterococcus faecium, Escherichia coli, Klebsiella pneumoniae, Mycobacterium tuberculosis, Pseudomonas aeruginosa*, and *Staphylococcus aureus*, reached their lowest levels in August 2024, peaked in November (winter), and declined again by March 2025, indicating pronounced seasonal fluctuations of these pathogens within the PICU (Figure 5d).

### Spatial distribution characteristics of ARGs across ICUs and sampling areas

**Figure 6:**
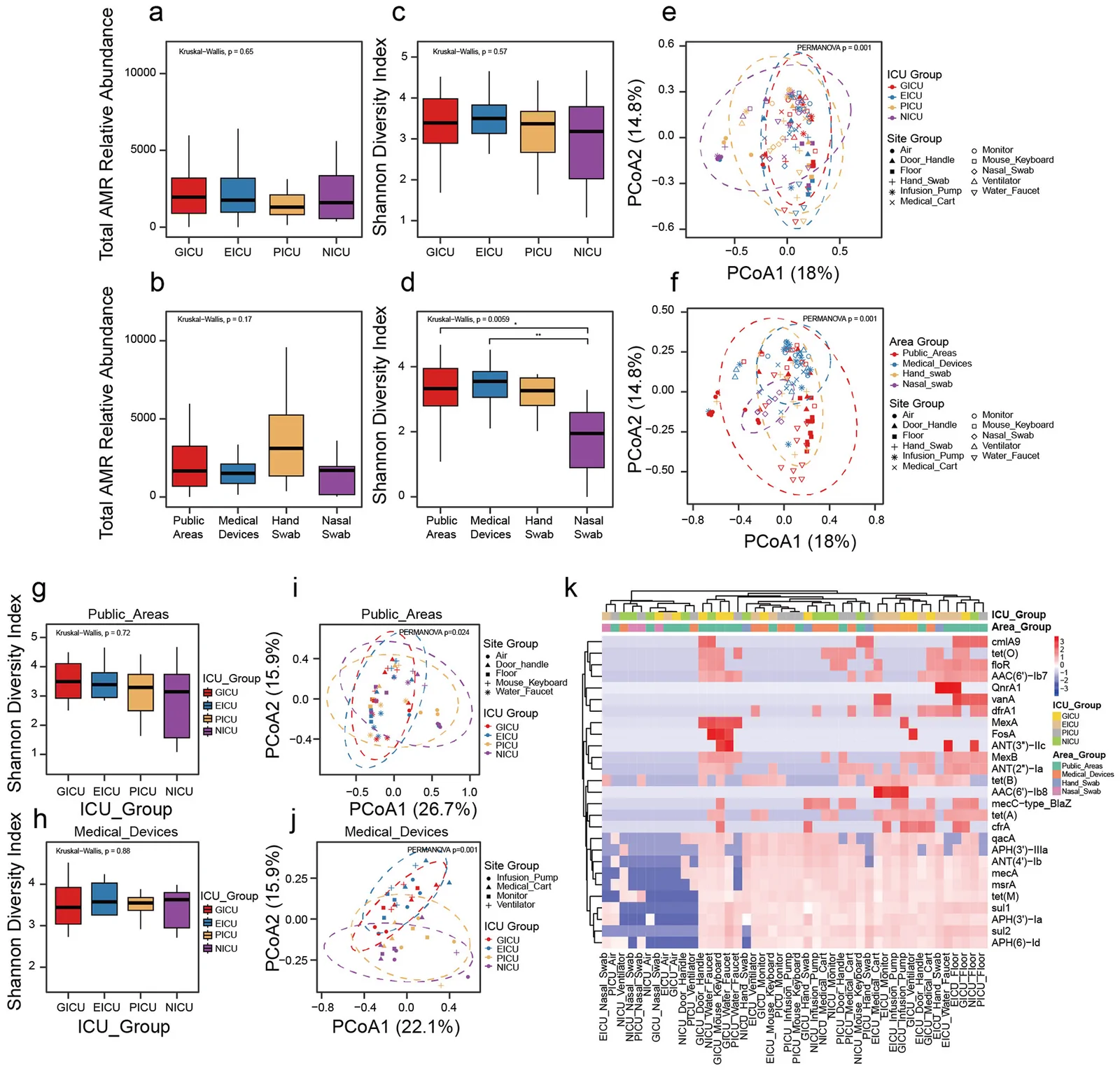
Spatial patterns of antimicrobial resistance genes (ARGs) across different ICUs type and sampling areas. **(a)** The total relative abundance of ARGs in GICU, EICU, PICU, and NICU. **(b)** The total relative abundance of ARGs in distinct ICU sampling areas (public areas, medical devices, nasal swabs, and hand swabs). **(c)** Alpha-diversity based on Shannon index of ARGs in GICU, EICU, PICU, and NICU. **(d)** Alpha-diversity based on Shannon index of ARGs in distinct ICU sampling areas. **(e)** PCoA of the beta diversity using Bray-Curtis distances of ARGs in GICU, EICU, PICU, and NICU. **(f)** PCoA of the beta diversity using Bray-Curtis distances of ARGs in distinct ICU sampling areas. **(g, h)** Alpha-diversity based on Shannon index of ARGs in public areas and medical devices among different ICUs. **(i, j)** PCoA of the beta diversity using Bray-Curtis distances of ARGs in public areas and medical devices among different ICUs. **(k)** The hierarchical clustering heatmaps of key ARGs associated with HAIs across different ICUs and sampling areas. *p < 0.05, **p < 0.01.

We first assessed the relative abundance of ARGs across different ICUs and sampling areas. No statistically significant differences in ARG relative abundance were observed among the four ICUs (Figure 6a). Among sampling areas, hand swab samples exhibited numerically higher ARG relative abundance compared to public areas, medical devices, and nasal swabs, though these differences did not reach statistical significance (Figure 6b).

For α diversity analysis, Shannon indices showed no significant differences in ARG diversity among the four ICUs (Figure 6c). In contrast, nasal swabs exhibited significantly lower α-diversity compared to public areas, medical devices, and hand swabs (*p* < 0.05; Figure 6d). ARG profiles presented significant differences among different ICUs based on PCoA analysis (PERMANOVA, *p* = 0.001; Figure 6e). Similar to microbial community profiles, ARG profiles of GICU and EICU tended to cluster together, while PICU and NICU exhibit greater similarity in ARG profiles. PCoA also showed clear separation of ARG profiles among the four sampling areas (PERMANOVA, *p* = 0.001; Figure 6f). When comparing the same sampling area across different ICUs, α-diversity analysis results showed no significant differences in public areas and medical devices (Figures 6g, h). However, PCoA exhibited considerable variation of ARG composition among different ICUs both in public areas (PERMANOVA, *p* = 0.024; Figure 6i) and medical devices (PERMANOVA, *p* = 0.001; Figure 6j), where GICU/EICU were clearly separated from PICU/NICU (Figures 6i, j). Considering limited sample sizes, we did not conduct diversity analysis for nasal and hand swab samples across ICUs.

We further examined the distribution patterns of 27 ARGs commonly associated with healthcare-associated pathogens, including *mecA, vanA, MexA, QnrA1, tet(A), tet(B), tet(M), tet(O), dfrA1, mecC*-type *BlaZ, qacA, MexB, FosA, floR, cmlA9, msrA, sul1, sul2, cfrA, ANT(2*′′*)-Ia, ANT(3*′′*)-IIc, ANT(4*′*)-Ib, APH(3*′*)-Ia, APH(6)-Id, APH(3*′*)-IIIa, AAC(6*′*)-Ib7*, and *AAC(6*′*)-Ib8*. Cluster heatmap analysis results (Figure 6k) showed that samples from the same ICU or similar area tended to cluster together, reflecting shared ARG profiles. From the distribution characteristics of specific ARGs, *cmlA9, tet(O), floR, AAC(6*′*)-Ib7, tet(A), tet(B), MexB*, and *ANT(2*′′*)-Ia* exhibited higher abundance in public area samples from GICU and EICU. Some genes, including *QnrA1, FosA, ANT(3*′′*)-IIc*, and *AAC(6*′*)-Ib8*, were only detected in a few samples. However, *vanA, dfrA1*, and *MexA* were presented at relatively high levels in some ICU samples, but were not widely distributed across all samples. Notablly, genes in the lower region of the heatmap, including *ANT(4*′*)-Ib, mecA, msrA, tet(M), sul1, sul2, APH(3*′*)-Ia*, and *APH(6)-Id*, were detected in most samples, though generally at low to moderate abundance. Moreover, we observed that among different sampling areas, these 27 ARG abundance in public area samples showed consistently higher level, whereas samples from nasal swabs and air exhibited lower ARG levels.

### Temporal variability of ARG profiles in the PICU

**Figure 7:**
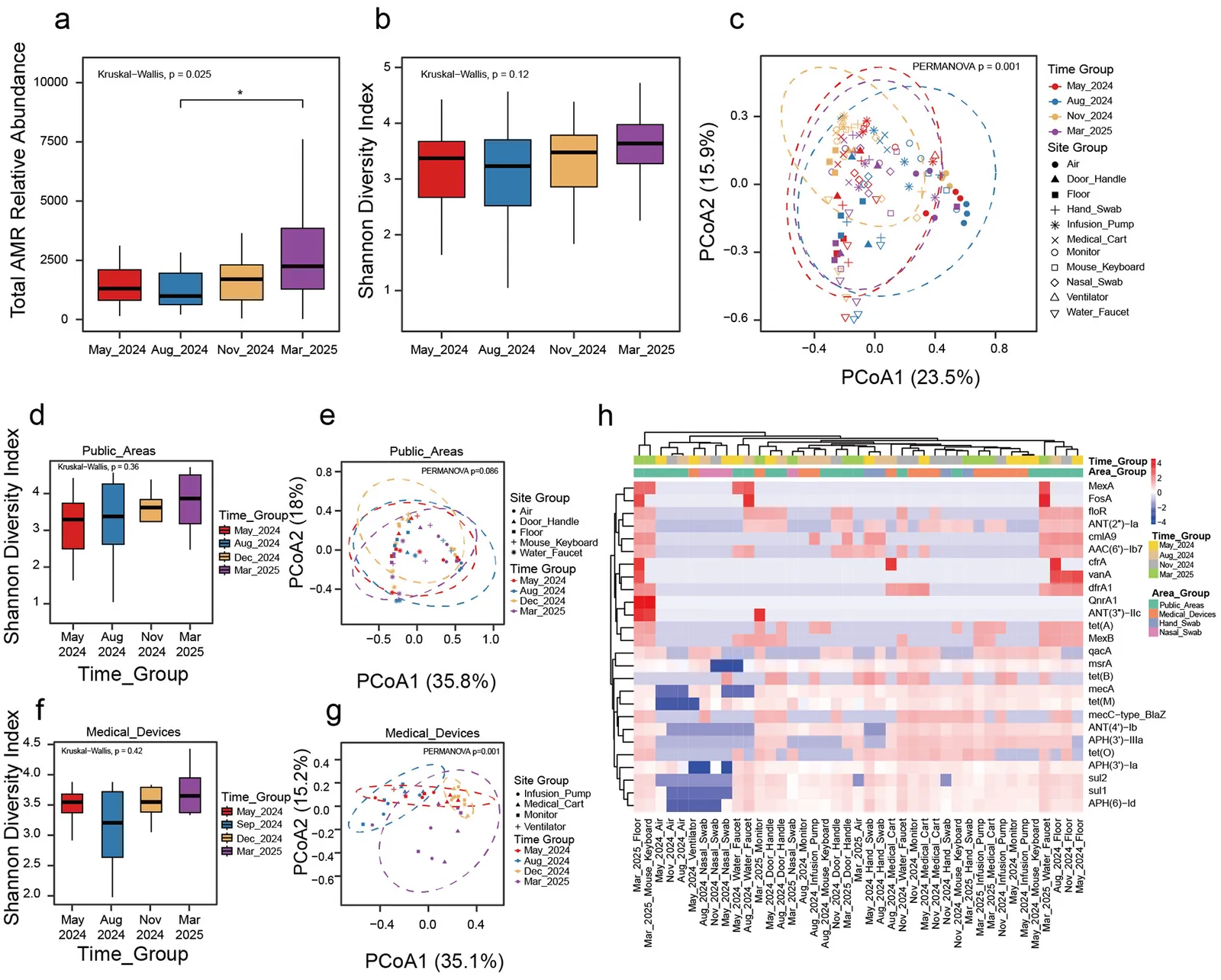
Temporal patterns of ARGs in the PICU over a 1-year period. **(a)** The total relative abundance of ARGs of different sampling time-points in PICU. **(b)** PCoA of the beta diversity using Bray-Curtis distances of ARGs of different sampling time-points in PICU. **(c)** PCoA of the beta diversity using Bray-Curtis distances of ARGs of different sampling time-points in PICU. **(d, f)** Alpha-diversity based on Shannon index of ARGs in public areas and medical devices among different sampling time-points in PICU. **(e, g)** PCoA of the beta diversity using Bray-Curtis distances of ARGs in public areas and medical devices among different sampling time-points in PICU. **(h)** The hierarchical clustering heatmaps of key ARGs associated with HAIs across different sampling time-points and sampling areas in PICU. *p < 0.05.

Analysis of the total relative abundance of ARGs across four time points in the PICU revealed clear temporal variation, with the lowest level in August 2024 and the highest in March 2025 (Figure 7a). The α diversity analysis showed no significant differences in ARGs diversity among different time points (Figure 7b), while PCoA revealed obvious temporal shifts in ARG composition (PERMANOVA, *p* = 0.001; Figure 7c). Both α diversity and PCoA analyses of public areas revealed no significant differences across the four time points (Figures 7d, e), indicating the ARG diversity and structure in public areas might be relatively stable over time. For the medical devices, no significant difference was also found in alpha diversity among the four time points (Figure 7f). However, ARG lineages in medical devices are grouped together according to when they were sampled (PERMANOVA, *p* = 0.001; Figure 7g).

Clustering heatmap analysis of key ARGs showed that the detection frequency and abundance of ARGs were higher in November 2024 and March 2025 (Figure 7h). Some genes, such as *QnrA1* and *ANT(3*′′*)-IIc*, were detected only in a subset of samples in March 2025. Across the different sampling areas, public areas showed the greatest accumulation of ARGs. The highest detection frequency of ARGs was found in floor samples, and air samples showed the lowest detection frequency. Several genes, such as *qacA, msrA, tet(M), ANT(4*′*)-Ib, APH(3*′*)-Ia, APH(3*′*)-IIIa, sul1, sul2*, and *APH(6)-Id*, were widely detected across various samples. *MexA, FosA, vanA*, and *dfrA1* were just detected in a few samples with high abundances. In addition, floors and door handles were mainly enriched with *floR, ANT(2*′′*)-Ia, cmlA9, tet(A), MexB*, and *AAC(6*′*)-Ib7*.

Association between environmental pathogen and ARG burden with clinical MDRO detection rates

**Figure 8:**
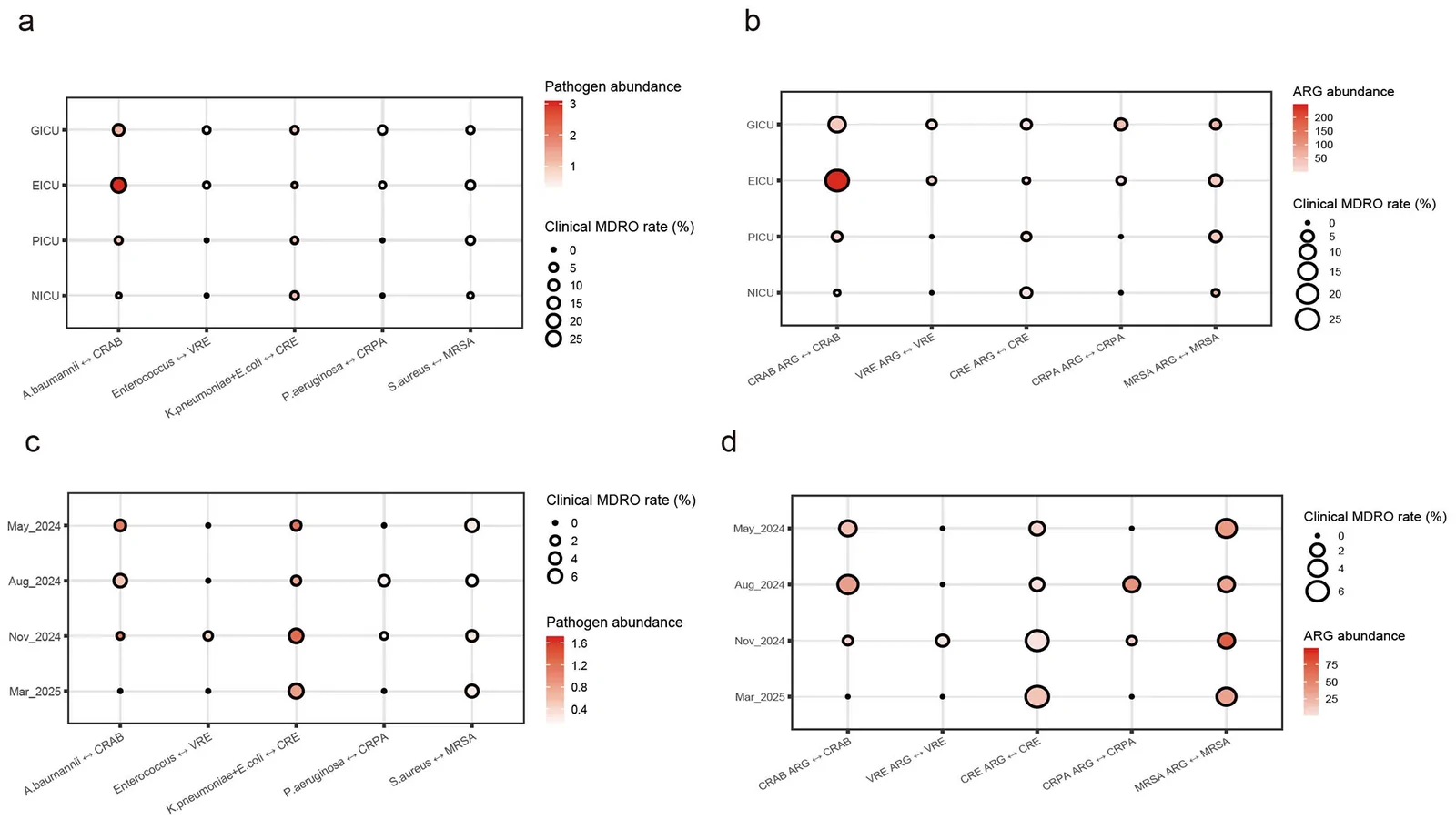
Comparison of environmental pathogen and ARG burden with clinical MDRO detection rates. **(a)** Environmental abundance of clinically relevant pathogens and corresponding MDRO detection rates across the four ICU. **(b)** ARG burdens associated with MRSA, CRE, CRAB, CRPA, and VRE compared with clinical MDRO detection rates across the four ICUs. **(c)** Temporal variation in environmental pathogen abundance and MDRO detection rates across different sampling time-points in PICU. **(d)** Temporal variation in aggregated ARG burdens and MDRO detection rates across different sampling time-points in PICU.

We compared the environmental abundance of clinically relevant pathogens and their corresponding ARGs with contemporaneous clinical MDRO detection rates across the four ICUs and four PICU sampling periods (Figure 8). Across ICUs, partial concordance was observed between environmental microbiological profiles and clinical MDRO detection rates (Figures 8a, b). The EICU exhibited the highest environmental abundance of *Acinetobacter baumannii* together with the highest CRAB detection rate. Likewise, CRAB-associated ARGs were markedly enriched in the EICU and showed a distribution consistent with clinical CRAB detection rates across ICUs. Environmental abundances of *Klebsiella pneumoniae* and *Escherichia coli* as well as VRE-associated ARGs were higher in the GICU and EICU, where VRE was also detected, whereas both indicators remained low in the PICU and NICU. In contrast, correspondence between environmental indicators and clinical detection rates was less evident for CRE, CRPA and MRSA.

Within the PICU, environmental pathogen abundance, ARG burden, and clinical MDRO detection rates all varied across the four sampling periods (Figures 8c, d). However, temporal changes in environmental indicators were not consistently reflected by corresponding MDRO detection rates. Elevated CRE detection rates were observed in both November 2024 and March 2025, whereas environmental abundances of *Klebsiella pneumoniae* and *Escherichia coli* remained comparatively moderate in March 2025. Similarly, several ARGs exhibited substantial temporal fluctuations that were not uniformly mirrored by changes in clinical MDRO detection rates.

## Discussion

Typically, multiple specialized ICUs are established in hospital to cater to the specific clinical care. The environmental microbial ecosystems in different ICUs may be unique due to the differences in patient demographics, clinical procedures, and physical layouts. Herein, the spatiotemporal changes of environmental microbiota and ARGs profiles of various ICUs were monitored using mNGS in a tertiary-care hospital in northwestern China.

This study found that α diversity was similar while β diversity was significantly different in microbial profiles across the GICU, EICU, PICU, and NICU. Interestingly, two distinct clustering patterns were observed between GICU/EICU and PICU/NICU. The similar observation is reported by a study of Brazil, which reveals that NICU and GICU exhibit different environmental microbial communities (Ribeiro et al., 2019). We also found that *Staphylococcus, Bacillus, Corynebacterium, Acinetobacter*, and *Moraxella* were dominant genera in ICU environments. Similar to our findings, previous ICU environmental surveillance studies conducted in China and other countries have reported comparable microbial profiles, characterized by the predominance of skin-associated bacteria such as *Staphylococcus* and *Corynebacterium* and healthcare-associated opportunistic pathogens including *Acinetobacter* and *Pseudomonas* (Cruz-López et al., 2023; Yang et al., 2023; Chopyk et al., 2020; Neag et al., 2025). Of note, skin commensals bacteria such as *Staphylococcus* and *Corynebacterium* as well as several HAI-associated pathogens, including *Acinetobacter baumannii, Klebsiella pneumoniae, Enterococcus faecalis*, and *Staphylococcus epidermidis*, were mainly enriched in GICU and EICU. In contrast, the PICU and NICU were dominated by respiratory-associated pathogens such as *Streptococcus pneumoniae* and *Burkholderia cepacia*. In terms of patient characteristics and clinical practice, GICU/EICU and PICU/NICU may exhibit significant differences in the colonization pressure of HAIs-associated pathogens. Patients in GICU/EICU are predominantly elderly individuals, with comorbidities of multi-organ dysfunction or malignancies, and often present with immunosuppression due to postoperative chemotherapy or long-term corticosteroid use (Chopyk et al., 2020; Blot et al., 2022). Moreover, the integrity of their skin and mucosal barriers may be compromised due to local damage caused by endotracheal intubation or central venous catheterization, which create favorable niches for the colonization of opportunistic pathogen (Blot et al., 2022). In addition, catheter-associated mucosal lesions also may facilitate biofilm formation by HAI-associated pathogens, such as *Acinetobacter baumannii, Staphylococcus epidermidis*, and *Klebsiella pneumoniae* (Gedefie et al., 2021; Nunez et al., 2023), ultimately contributing to persistent colonization and potential bloodstream infections (Gedefie et al., 2021; Nunez et al., 2023; Zhang et al., 2016). Patients in PICU/NICU predominantly present with single-system diseases and receive short-term life-support interventions (McMann et al., 2024). Compared with adult ICU, the interventions in PICU/NICU are both lower in intensity and shorter in exposure duration, thereby mitigating microbial colonization pressure (McMann et al., 2024). Therefore, GICU/EICU and PICU/NICU may require the implementation of targeted disinfection and infection prevention and control strategies.

Hospital environmental surfaces are considered as important medium for microbial transmission (Salazar et al., 2022). Previous studies have demonstrated that environmental surfaces, including tables, bed rails, monitor panels, ventilator buttons, stethoscopes, and central venous catheters, are enriched by various microorganisms (Cruz-López et al., 2020, 2021; Russotto et al., 2015). In this study, public areas and medical devices in ICUs were dominated by desiccation-resistant and disinfectant-resistant genera, including *Staphylococcus, Bacillus, Corynebacterium, Burkholderia*, and *Cutibacterium*. Hand swabs displayed a mixed microbial composition, including *Staphylococcus, Streptococcus, Corynebacterium*, and *Malassezia*. Nasal swabs exhibited the lowest diversity yet the most distinct community profile, with enrichment of clinically important pathogens such as *Acinetobacter baumannii, Staphylococcus aureus, Klebsiella pneumoniae, Streptococcus pneumoniae, Escherichia coli, Pseudomonas aeruginosa*, and *Mycobacterium tuberculosis*. An observational study has revealed that microbiota on hospital surfaces may reach the respiratory mucosa or even deeper tissue via airborne or contact transmission (Cason et al., 2021). Then, HAI-associated pathogens in the nasal cavity such as *Staphylococcus aureus* may migrate to the lower respiratory tract, thereby leading to ventilator-associated pneumonia (Rocha et al., 2013; Pickens and Wunderink, 2022). In this study, some fungi, such as *Candida albicans* and *Aspergillus fumigatus*, were also detected in ICU environments. We found that fungi in environmental samples exhibited higher abundance than those from patient, indicating that ICU environments may serve as persistent reservoirs for these fungi. Notably, GICU/EICU showed higher abundance of *Candida albicans* in comparison with PICU/NICU. This finding may be attributed to higher incidence of immunosuppression, prolonged antibiotic use, and frequent invasive procedures, all of which disrupt mucosal barriers and promote fungal colonization as well as biofilm formation. Supporting our findings, an mNGS-based environmental microbiome monitoring study also confirmed that *Candida albicans* is highly enriched in the geriatric ICU environment (Yang et al., 2023). These results indicate that different ICU environments, from inanimate surfaces to human mucosal sites, experience distinct selective pressures that together create a gradient in microbial colonization.

Furthermore, longitudinal monitoring of the PICU environment over 1 year revealed that both the environmental and patient-associated microbial communities in the PICU exhibited significant dynamic changes on an annual timescale. Previous study has shown that temperature and climate can influence microbial communities in built environments (Lim et al., 2021). Li et al. (2022) reported that in GICU, both microbial α-diversity and community composition varied significantly across a 12-month period. In contrast, our study found no significant seasonal differences in α-diversity within the PICU, whereas β-diversity analysis revealed clear temporal separation of community structures, suggesting that the composition rather than the richness of the microbial community fluctuated across seasons. This discrepancy may stem from differences in ICU types, as the GICU studied by Li et al. and the PICU examined in our study differ in patient disease profiles, immune status, and microbial colonization potential, which may influence the temporal stability of microbial α-diversity. Previous studies have clearly demonstrated that seasonal variations can influence the composition of microbial communities in the environment (Lax et al., 2017; Lax and Gilbert, 2015; Ramos et al., 2015). Similarly, this study showed that time had a pronounced effect on the microbial composition of public areas and medical device surfaces than on patient-derived samples. This discrepancy may be related to different microbial regulation mechanisms between the environment and the human body. Furthermore, the major pathogens, such as *Acinetobacter baumannii, Klebsiella pneumoniae, Pseudomonas aeruginosa*, and *Staphylococcus aureus*, exhibited the highest abundance in winter. Although seasonal variation was observed, the present study did not include measurements of environmental variables, such as ventilation, temperature, humidity, or other indoor air quality parameters. Therefore, the mechanisms underlying the observed winter increase cannot be determined. Future studies integrating environmental monitoring with metagenomic surveillance will be valuable for elucidating the drivers of seasonal microbial dynamics in ICU environments. Nevertheless, our findings indicate that seasonal variation should be considered when designing ICU environmental surveillance and infection prevention strategies.

The transmission of ARGs is also a serious challenge for drug-resistant pathogen infections in ICU environments (Ahmad et al., 2021). It has been demonstrated that the accumulation and transmission of ARGs were contributed by intensive invasive procedures, frequent antibiotic usage, and high mobility of healthcare workers and patients (Salam et al., 2023; Mikolas et al., 2025). In addition, from a One Health perspective, ICU environmental antimicrobial resistance cannot be interpreted independently of extra-hospital ecological reservoirs, among which migratory wild birds represent a high-risk mobile vector driving intercontinental ARG dissemination, as systematically demonstrated by a recent nationwide metagenomic study of wild migratory microbiomes across China (Wang et al., 2026). Consistent with microbial profiles, a clear separation was also observed in ARGs profiles between GICU/EICU and PICU/NICU samples. In addition, ARGs showed the highest abundance in high-touch environmental surfaces, such as floors and doorknobs, while a lower level in air and nasal swab samples. This observation confirms that environmental surfaces in ICUs are primary reservoirs for ARGs. The detected ARGs in this study spanned a broad range of antibiotic categories, including resistance determinants to β-lactams (*mecA, mecC, BlaZ*), glycopeptides (*vanA*), fluoroquinolones (*QnrA1*), tetracyclines (*tet series*), aminoglycosides (*ANT, APH, AAC* series), sulfonamides (*sul1, sul2*), chloramphenicol (*cmlA9, floR*), macrolides (*msrA*), fosfomycin/fluoroquinolone (*FosA*), and antiseptics (*qacA*). These ARGs involve various resistance mechanisms, such as hydrolysis, efflux pumps, target modification, and enzymatic drug modification (Tang et al., 2023). Many of them were closely linked to pathogens frequently implicated in HAIs: *mecA*/*mecC*/*BlaZ* with MRSA (Pimenta et al., 2023); *vanA* with *Enterococcus spp*. (VRE) (Liu et al., 2022); *QnrA1* with fluoroquinolone-resistant *Klebsiella spp*. and *Pseudomonas aeruginosa* (Kawa et al., 2023); *tet* genes with *Staphylococcus, Enterococcus*, and some Gram-negative bacteria (Chopra and Roberts, 2001); *ANT, APH, AAC* series with *Pseudomonas aeruginosa, Acinetobacter baumannii*, and *Enterobacteriaceae* (Zhang et al., 2023). Specifically, β-lactam ARGs (*mecA*) were widely distributed in most samples due to dominance of MRSA and *Pseudomonas aeruginosa*. Tetracycline ARGs [*tet(A), tet(B)*] were locally enriched in adult ICU public areas, while aminoglycoside ARGs [*ANT(2*′′*)-Ia, AAC(6*′*)-Ib7*] showed broad distribution. Conversely, *vanA* and *QnrA1* were less prevalent and appeared sporadically, reflecting lower colonization rates of their host bacteria and restricted antibiotic usage. Temporally, ARG abundance increased from November 2024 to March 2025, and genes such as *QnrA1* and *ANT(3*′′*)-IIc* were only detected in March 2025, possibly linked to seasonal peaks in respiratory and gastrointestinal infections and corresponding antibiotic use. These results indicate that ARG profiles exhibit considerable spatial and temporal variation in the PICU. Furthermore, our results demonstrated that the profiles of resistance genes detected in samples from various ICUs, especially water faucet sink samples, were highly consistent with those carried by pathogens in hospital wastewater reported in previously published studies. This phenomenon suggests persistent circulation of resistance genes and drug-resistant strains between ICU hand sinks and hospital drainage systems, and the aquatic environment in ward areas may serve as a critical reservoir facilitating the horizontal transmission of multidrug-resistant bacteria (Chen et al., 2025).

To further explore the clinical relevance of the environmental data, we compared environmental pathogen and ARG burden with contemporaneous MDRO surveillance results. Several spatial patterns observed in the environmental data were reflected in the clinical surveillance records, particularly for CRAB and VRE. However, comparable temporal relationships were not consistently observed within the PICU. Given the complex determinants of MDRO transmission and detection, including patient-related and infection control factors, such discrepancies are not surprising. Together, these findings suggest that environmental metagenomic surveillance may complement conventional infection control monitoring by providing additional insight into the ecological reservoirs of ARGs in ICU environments.

Several limitations should be considered when interpreting our findings. Only the PICU was sampled longitudinally, whereas the other ICUs were surveyed at a single time point. Cluster heatmaps results (Figure 5a) showed that most key pathogens exhibited higher relative abundances in adult ICUs (GICU and EICU) compared with PICU /NICU. Accordingly, the temporal dynamics observed within the PICU may not represent the patterns present in other ICU environments. Notably, children admitted to the PICU possess immature immune function, a unique spectrum of infectious diseases, distinct antibiotic exposure profiles, and are managed under different environmental protocols relative to adult ICU patients. Such discrepancies in patient populations and clinical practices may drive divergent seasonal dynamics of pathogens and antimicrobial resistance genes. We also included contemporaneous MDRO surveillance data to provide clinical context for the environmental observations. However, patient-level epidemiological information, isolate collections, and genomic linkage data were not available, making it impossible to directly assess transmission between environmental reservoirs and clinical cases. Importantly, positive environmental detection of MDROs therefore cannot be interpreted as definitive evidence of ongoing transmission events. Environmental contamination only demonstrates the existence of a potential pathogen reservoir, and strain-level genomic comparison is required to confirm clonal relatedness between environmental isolates and patient-derived strains. This inherent disconnect between environmental surveillance results and verified clinical transmission represents a key limitation of the present study. In addition, metagenomic sequencing detects microbial DNA and resistance determinants regardless of viability. The method cannot distinguish DNA derived from viable infectious cells from residual DNA of dead bacteria or free extracellular DNA in the environment. The presence of a pathogen or ARG in the environment should not be interpreted as evidence of active transmission. The identified genetic material may not correspond to microorganisms capable of colonizing or infecting patients. Further investigations incorporating bacterial culture are needed to validate the viability and actual transmission potential of these environmental microbes (Emerson et al., 2017).

## Conclusions

In summary, our study preliminarily described the spatiotemporal patterns of microbial and ARG profiles in ICU environments. Both microbial and ARG profiles exhibited clear two-group clustering tendencies between GICU/EICU and PICU/NICU. Several HAI-associated pathogens, such as *Acinetobacter baumannii, Pseudomonas aeruginosa, Staphylococcus aureus, Klebsiella pneumoniae*, and *Streptococcus pneumoniae*, were simultaneously existed in environmental surfaces, hand swabs, and nasal swabs, and the relative abundances of these pathogens were highest in winter. In addition, ARGs also widely presented in environmental surfaces. Thus, the disinfection strategies should be customized based on the microbial characteristics of different ICUs types, time periods, and environmental areas. This study improved our understanding of the transmission dynamics of pathogens and ARGs in distinct ICUs, providing new insights into the prevention and control of HAIs.

## Data Availability

The datasets presented in this study can be found in online repositories. The names of the repository/repositories and accession number(s) can be found below: https://ngdc.cncb.ac.cn/gsa/browse/CRA041360, CRA048943, PRJCA054971.
