# Supplementary material for: Spatiotemporal characteristics of environmental microbial communities and resistomes in intensive care units of a tertiary hospital in northwest China
Source: Front Microbiol. 2026 Sep 9;17:1909107. doi: 10.3389/fmicb.2026.1909107 (PMC13597873; doi:10.3389/fmicb.2026.1909107)
Supplement: Supplementary file 1 [file Table_1.docx]

**Supplementary Information**

**Supplement Figure 1 Microbial community characteristics of the same sampling areas across different ICUs.** a, b and c. Alpha-diversity based on Shannon index at identical sampling areas (including public areas, medical devices, nasal swabs, and hand swabs) across different ICUs. d ,e and f. PCoA of the beta diversity using weighted Unifrac distances at identical sampling areas across different ICUs. g, h and i. The mean relative abundances of top 20 genera at identical sampling areas across different ICUs.
